# Supplementary material for: Cis-regulatory architecture of human ESC-derived hypothalamic neuron differentiation aids in variant-to-gene mapping of relevant complex traits
Source: Nat Commun. 2021 Nov 19;12:6749. doi: 10.1038/s41467-021-27001-4 (PMC8604959; doi:10.1038/s41467-021-27001-4)
Supplement: Supplementary file 1 — Supplementary Information [file 41467_2021_27001_MOESM1_ESM.pdf]

Supplementary Information for Cis-regulatory architecture of human ESC-derived hypothalamic neuron differentiation aids in variant-to-gene mapping of relevant complex traits

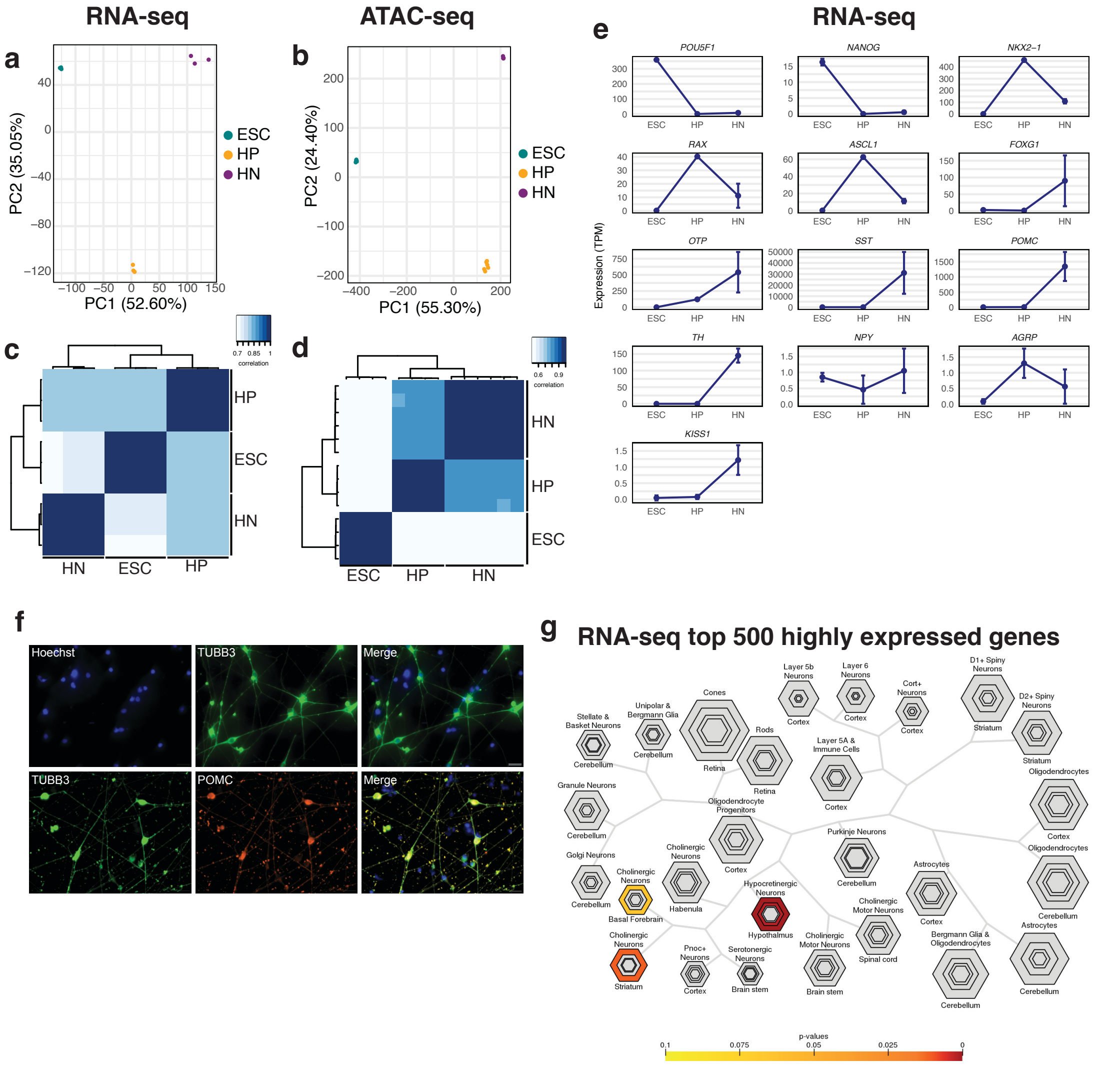

Supplemental Figure 1: Validation of ESC derived hypothalamic neurons gene expression signature. (A) The first and second principal components of RNA-seq and (B) ATAC-seq samples. The variance explained by each principal component is indicated on each axis. (C) Pairwise Pearson correlation coefficients of RNA-seq and (D) ATAC-seq samples. (E) The expression (TPM) of selected marker genes during the three stages of differentiation. (F) Differentiated hypothalamic neurons were stained with the neuronal marker TUBB3 and the

neuropeptide marker POMC. Hoechst stain identifies nuclei. In the upper row from left to right TUBB3 (secondary 488), Hoechst (blue) and Merge. In the lower row from left to right TUBB3 (secondary 488), POMC (secondary 555) and Merge. Scale bar 100 μm. (F) Cell specific enrichment analysis of the top 500 expressed genes in HN against a set of marker genes from various different central nervous system neuronal and glial cell types. Rings indicate stringency of marker gene cell specificity (outer = liberal, inner = stricter) Color indicates statistical significance.

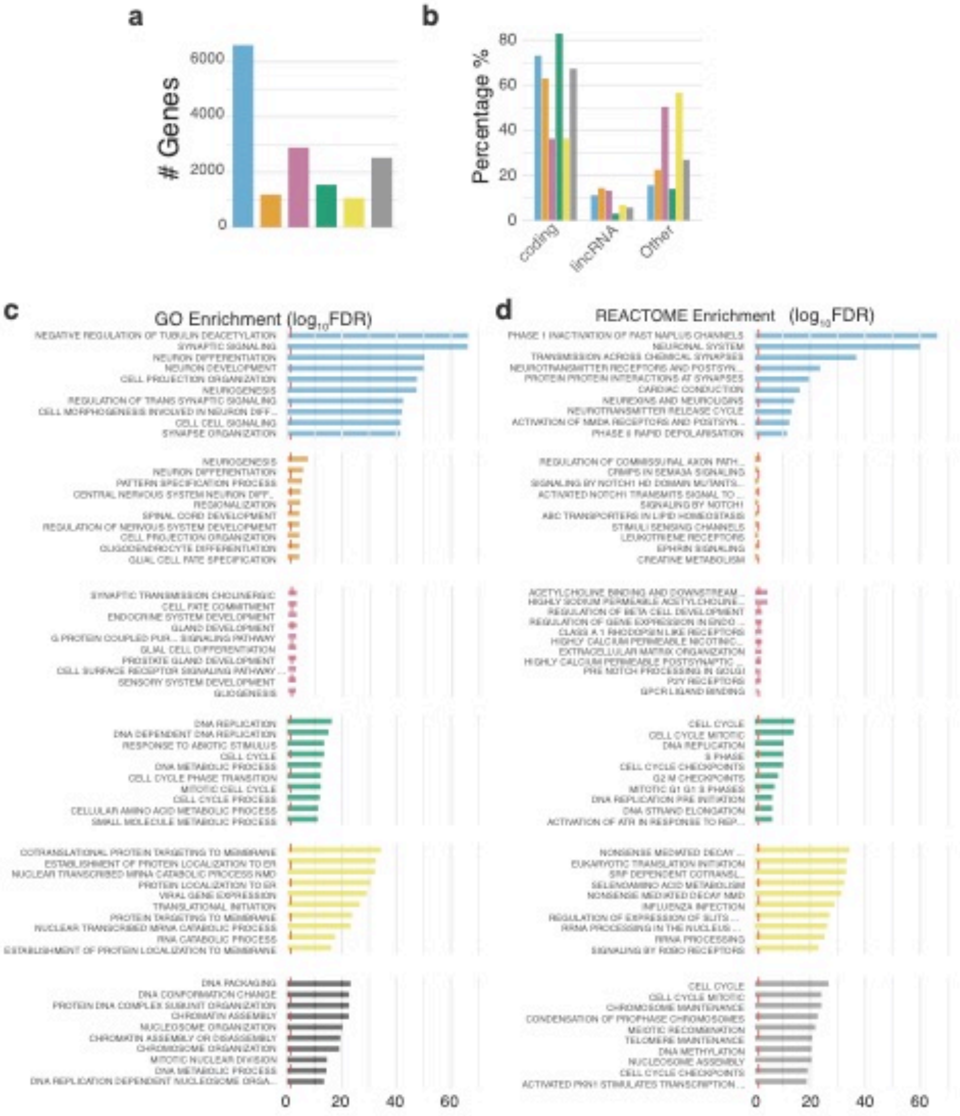

Supplemental Figure 2: Summary and functional annotation of expression clusters. (A) Counts of the number of genes assigned to each cluster. (B) The relative number of genes annotated as either protein coding, lincRNA, or other annotation (e.g. pseudogenes). (C) Top 10 GO term or (D) REACTOME pathway enrichment of each gene group. The bar's length depicts the -log10 transformed FDR adjusted p-values. The red dotted line indicates the threshold for statistical significance (FDR < 0.05).

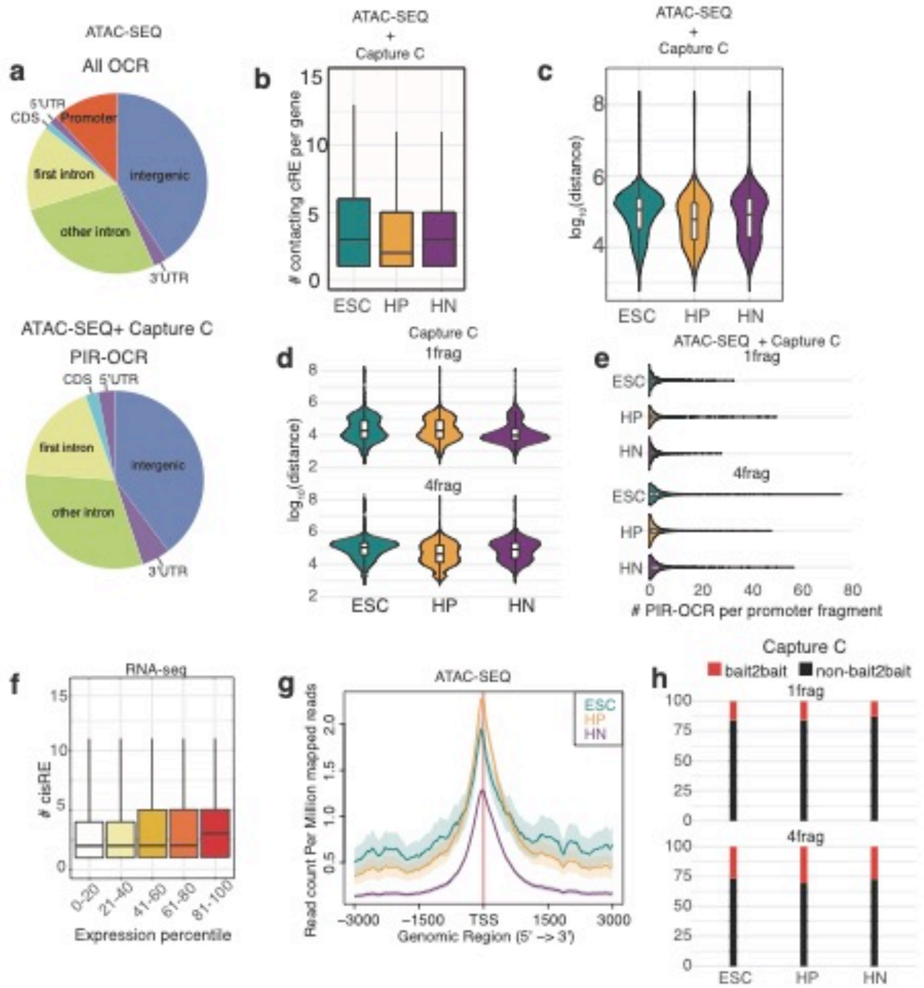

Supplemental Figure 3: Genomic features of promoter connected OCRs. (A) The genomic location of all OCRs and the (C) PIR-OCRs. (B) Distribution of the number of cRE at each stage. (C) Quantification of the distance (log10) of each cRE (D) Distance between fragments detected by Promoter-focused Capture C at 1-fragment and 4-fragment resolutions from each stage of differentiation. (E) Distribution of the number of PIR-OCRs linked to each gene in each stage of differentiation. (F) The distribution of the number of cRE binned by expression quantile. (G) Chromatin accessibility profile relative to all transcription start sites. (H) Percentage of fragments with interactions to other promoter bait fragments (bait-to-bait) at each resolution.

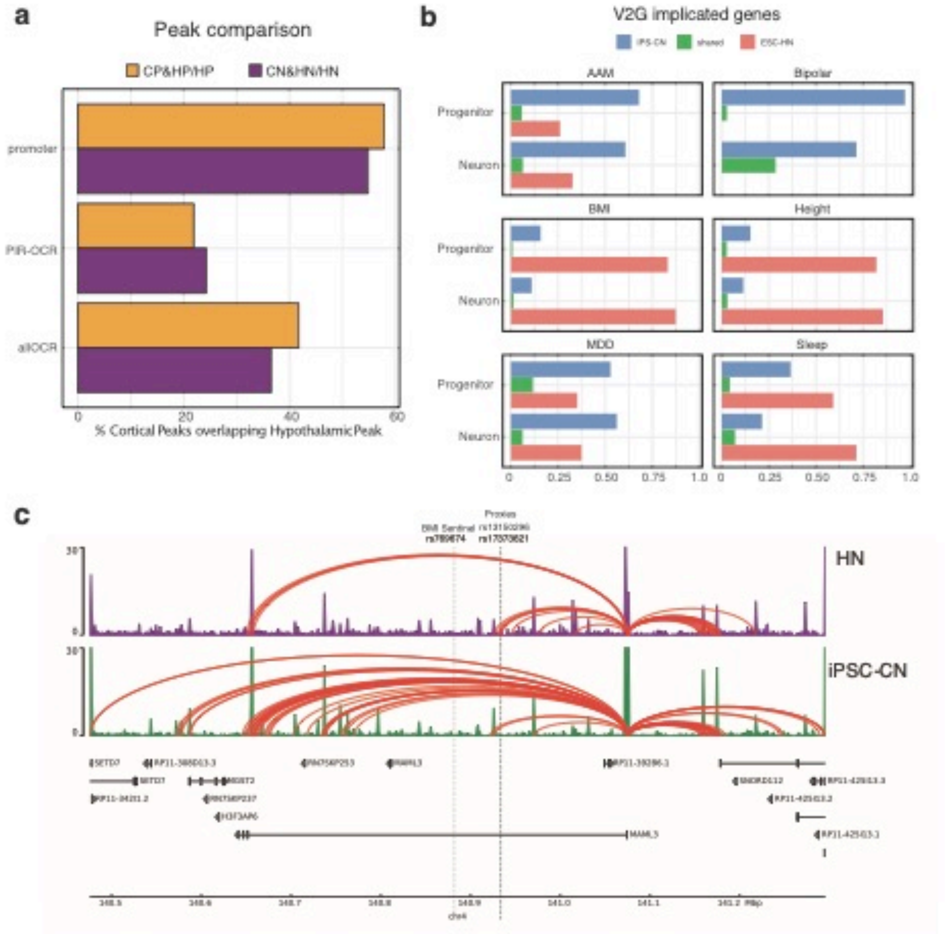

Supplemental Figure 4: Comparison of cRE with epigenetic data from mouse hypothalamus. We compared the cREs identified in HNs with prior enhancers (ATAC-seq + H3K27ac) identified from sorted leptin receptor positive and negative hypothalamic neurons from mice. (A) We performed permutation testing and found that HN were enriched for enhancer regions identified in mice (liftover of coordinates from mm9 to hg19). (B) A set of 4030 H3K27ac peaks were enriched in leptin-receptor positive neurons across conditions. Approximately 29% of leptin-receptor positive H3K27ac peaks with our set of promoter-connected OCRs. There were 1,542 connected genes to these OCRs with 647 (42%) of them with TPM > 1. (C) Example genomic

tracks for HN cRE overlapping with mouse leptin-receptor positive hypothalamic neuron enriched H3K27ac peaks for *ISL1* (C) and *CRH* (D).

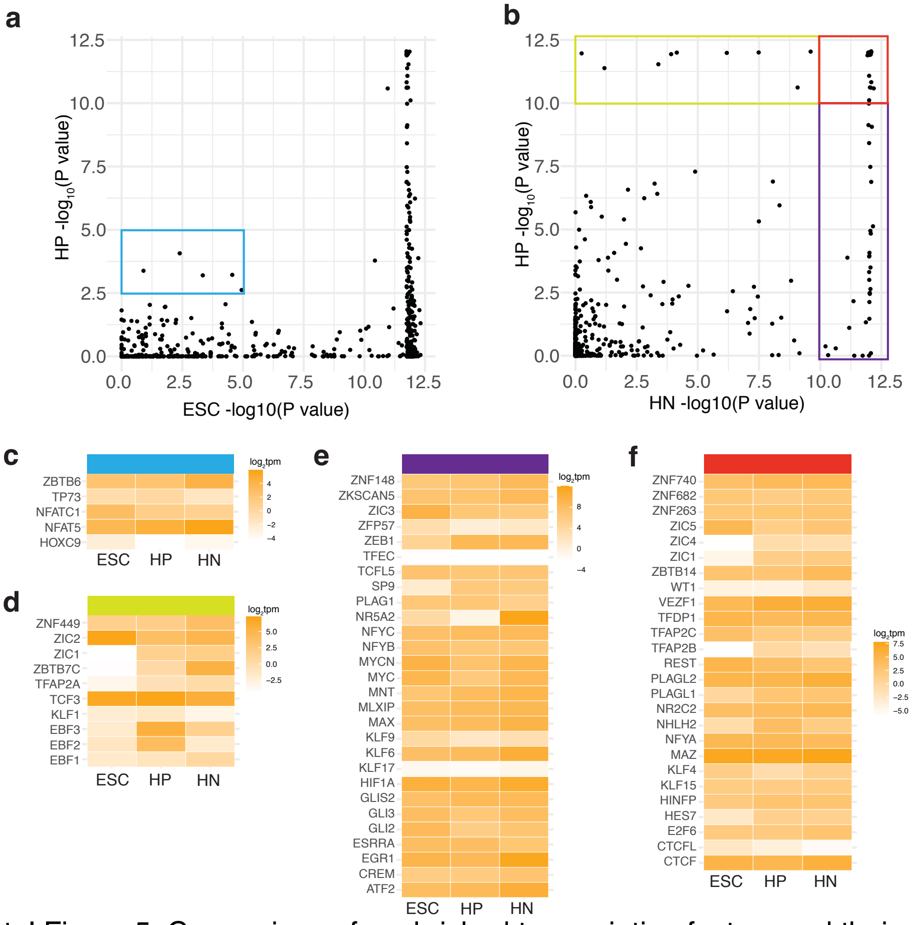

Supplemental Figure 5: Comparison of enchriched transcription factors and their expression. (A) Comparison between ESC and HP enrichment scores (-$\log_{10}$(BiFET P value)). (B) Comparison between HN and HP enrichment scores. (C-F) Expression ($\log_2$TPM) of top enriched TFs biased toward HPs compared to ESC (C), HP compared to HN (D) and HN to HP (E), and TFs highly enriched in both HPs and HNs (F).

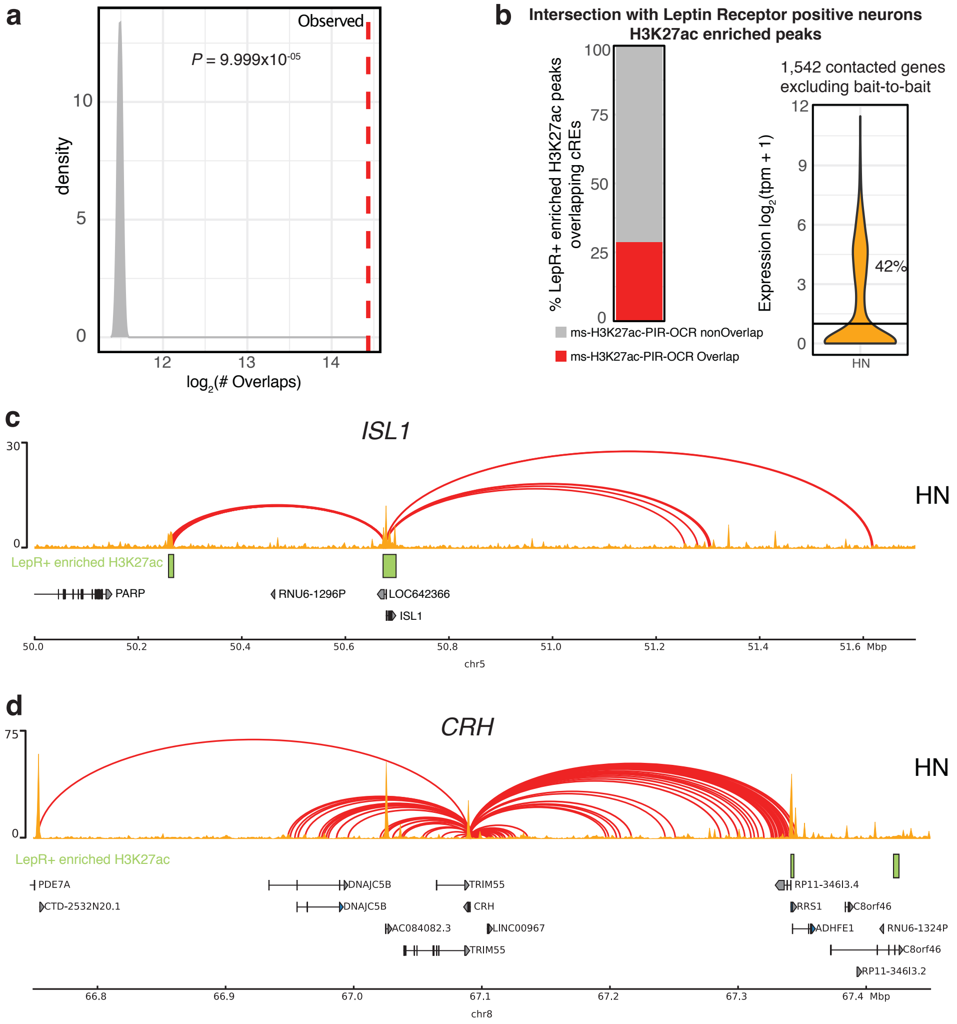

Supplemental Figure 6: Comparison between the OCRs, PIR-OCRs, and promoter OCRs between HP & HN this study and iPSC derived neurons dataset[53]. (A) The percentage of HP peaks that overlap with a cortical peak in each category. Comparison of the genes implicated by variant to gene mapping of the two datasets. (C) Example locus with BMI associated open proxy contacting the *MAML3* promoter in HNs but not iPSC derived neurons.
