# Supplementary material for: Cis-regulatory architecture of human ESC-derived hypothalamic neuron differentiation aids in variant-to-gene mapping of relevant complex traits
Source: Nat Commun. 2021 Nov 19;12:6749. doi: 10.1038/s41467-021-27001-4 (PMC8604959; doi:10.1038/s41467-021-27001-4)
Supplement: Supplementary file 3 — Description of Additional Supplementary Files [file 41467_2021_27001_MOESM3_ESM.pdf]

Description of Additional Supplementary Files

File Name: Supplementary Data 1
Description: Results from Spearman Correlation tests between HNs and GTeX tissues.Tests represent two-sided Spearman Rank Correlation.

File Name: Supplementary Data 2
Description: Enriched TF motifs in cRE compared to unannotated OCRs

File Name: Supplementary Data 3
Description: Results for partitioned LD score regression for the indicated genotypes.

File Name: Supplementary Data 4
Description: DAVID annotations of implicated genes for each trait.

File Name: Supplementary Data 5
Description: Gene annotation to each indicated proxy and sentinel SNP.

File Name: Supplementary Data 6
Description: Enriched GO terms of genes implicated by our V2G pipeline mapping specifically in HPs and HNs. P values were adjusted with FDR.

File Name: Supplementary Data 7
Description: Overlap between GWAS traits, 0 = not implicated, 1 = implicated.

File Name: Supplementary Data 8
Description: Annotation of SNPs located in TF binding sites.

File Name: Supplementary Data 9
Description: List of reagents and software used in this study.
