# Supplementary material for: Cis-regulatory architecture of human ESC-derived hypothalamic neuron differentiation aids in variant-to-gene mapping of relevant complex traits
Source: Nat Commun. 2021 Nov 19;12:6749. doi: 10.1038/s41467-021-27001-4 (PMC8604959; doi:10.1038/s41467-021-27001-4)
Supplement: Supplementary file 13 — Reporting Summary [file 41467_2021_27001_MOESM13_ESM.pdf]

nature portfolio

Corresponding author(s): Struan F.A. Grant / NCOMMS-20-39829B

Last updated by author(s): Sep 21, 2021

Reporting Summary

Nature Portfolio wishes to improve the reproducibility of the work that we publish. This form provides structure for consistency and transparency in reporting. For further information on Nature Portfolio policies, see our Editorial Policies and the Editorial Policy Checklist.

Statistics

For all statistical analyses, confirm that the following items are present in the figure legend, table legend, main text, or Methods section.

n/a	Confirmed	
☐	☒	The exact sample size (*n*) for each experimental group/condition, given as a discrete number and unit of measurement
☐	☒	A statement on whether measurements were taken from distinct samples or whether the same sample was measured repeatedly
☐	☒	The statistical test(s) used AND whether they are one- or two-sided<br>*Only common tests should be described solely by name; describe more complex techniques in the Methods section.*
☐	☒	A description of all covariates tested
☐	☒	A description of any assumptions or corrections, such as tests of normality and adjustment for multiple comparisons
☐	☒	A full description of the statistical parameters including central tendency (e.g. means) or other basic estimates (e.g. regression coefficient) AND variation (e.g. standard deviation) or associated estimates of uncertainty (e.g. confidence intervals)
☐	☒	For null hypothesis testing, the test statistic (e.g. *F*, *t*, *r*) with confidence intervals, effect sizes, degrees of freedom and *P* value noted<br>*Give P values as exact values whenever suitable.*
☒	☐	For Bayesian analysis, information on the choice of priors and Markov chain Monte Carlo settings
☒	☐	For hierarchical and complex designs, identification of the appropriate level for tests and full reporting of outcomes
☐	☒	Estimates of effect sizes (e.g. Cohen's *d*, Pearson's *r*), indicating how they were calculated

Our web collection on statistics for biologists contains articles on many of the points above.

Software and code

Policy information about availability of computer code

Data collection	Illumina bcl2fastq2 conversion v2.29
Data analysis	ATAC-seq<br>ATAC-seq peaks were called using the ENCODE ATAC-seq pipeline (https://github.com/kundajelab/atac_dnase_pipelines). Briefly, pair-end reads from each replicate for the cellular stages were aligned to hg19 genome using bowtie2 (v 1.1.2), and duplicate reads were removed prior to peak calling. Narrow peaks were called independently for each replicate using macs2 (v 2.1.0.20150731; -p 0.01 --nomodel --shift -75 -- extsize 150 -B --SPMR --keep-dup all --call-summits) and ENCODE blacklist regions (ENCSR636HFF) were removed from peaks in individual replicates. Peaks from all replicates were merged by bedtools (v2.25.0) within each cell type and the merged peaks present in less than half of biological replicates were removed from further analysis. Finally, ATAC-seq peaks from the three types were merged to obtain reference open chromatin regions.<br><br>Statistical analyses were performed using R (v 3.2.2). Quantitative comparisons of ESC, HP, and HN chromatin accessibility were performed by evaluating read count differences against the reference OCR set. De-duplicated read counts for OCR were calculated for each library and normalized against background (10K bins of genome) using the R package csaw (v 1.8.1). OCR peaks with less than 1.2 CPM support in the top third of samples were removed from further differential analysis. Differential analysis was performed independently using edgeR (v 3.16.5). Differential OCR between cell types were called if FDR < 0.05 and abs(log2 FC) > 1 in both methods.<br><br>Genomic Annotations: Promoters were defined as 1500kb upstream and 500kb downstream of the TSS (Genecode V19). Overlapping annotations were assigned to genomic features based on a hierarchy of (1) Promoter, (2) 5'UTR, (3) CDS, (4) 3'UTR, (5) first intron, (6) other introns, or (7) intergenic. These overlaps were done using the R package GenomicRanges (v1.26.4). The percentage of OCRs overlapping with each feature was visualized as pie charts using ggplot2 v3.3.0.<br><br>RNA-seq

Paired-end Fastq files with for each replicate were mapped to the reference genome (hg19) using STAR (v2.6.0c). Gene features were assigned to a curated annotation consisting of GencodeV19 with lincRNA and sno/miRNA annotation from the UCSC Table Browser. The raw read count for each gene feature was calculated using HTSeq-count function from HTseq (v0.6.1). with parameter settings -f bam -r pos -s reverse -t exon -m intersect. The genes located on chrM or annotated as ribosomal RNAs were removed before further processing.

Differential analysis was performed in R using the edgeR package (v3.16.5). Briefly, the raw reads per genes features were converted to read Counts Per Million mapped reads (CPM). The gene features with median value of less than 0.7 CPM (10 ~ 18 reads per gene feature) across all samples were filtered. Normalization scaling factors were calculated using the trimmed mean of M-values method. Differentially expressed genes between ESCs, HPs, and HNs were identified with thresholds of FDR < 0.05 and absolute log2FC > 1. Expression values are reported as transcript per million mapped reads (TPM). We clustered standardized TPM values of differentially expressed genes using the R function hclust. Genes expression values were standardized using the R function, scale. Following this, the top six branches were cut to define the clusters used in subsequent comparisons.

GO and REACTOME datasets annotated in MSigDB (v7.0) were used for gene set enrichment analyses. Statistical significance of gene set enrichment was determined using the hypergeometric test, implemented in the R phyper function.

Promoter Focused Capture C
For Capture-C analysis, paired-end reads from three replicates for each celltype were pre-processed using the HICUP pipeline (v0.5.9) {Wingett:2015}, with bowtie2 as the aligner and hg19 as the reference genome. Significant promoter interactions at 1-DpnII fragment resolution were called using CHiCAGO (v1.1.8) with default parameters except for binsize set to 2500, to account for differences in background estimation for 4cutter design. Significant interactions at 4-DpnII fragment resolution were also called using CHiCAGO with artificial. baitmap and .rmap files in which DpnII fragments were concatenated in silico into 4 consecutive fragments using default parameters except for removeAdjacent set to False. The significant interactions (CHiCAGO score > 5) from both 1-fragment and 4-fragment resolutions were exported in .ibed format.

Transcription factor analysis
PIQ, which integrates TF motif scanning with TF footprinting using DNAase or ATAC-seq data, was used to predict TF binding sites. We scanned the set of JASPAR2020 core PWMs against hg19, with ENCODE blacklist regions excluded using the default settings. For downstream analyses we considered TF binding sites passing the default cutoff of purity > 0.7. We identified TF motifs enriched in cREs compared the set of nonPIR-OCRs using the R package BiFET (v 1.4.0), with a cutoff of FDR<0.05,

Partitioned LDscore regression
Partitioned heritability was measured using LD Score Regression v1.0.0. Partitioned LDSR requires the GWAS summary statistics and a feature annotation. ESC, HP, and HN annotations were generated using bed files containing positions of the cRE (promoter OCRs + PIR-OCRs) with +/-500bp extension. We used baseline phase LD reference provided at https://github.com/bulik/ldsc.We selected a set of traits related to metabolic, endocrine, and neuropsychiatric traits with available GWAS summary statistics. Only summary statistics from EUR ancestries were used when applicable. Summary stats did not include data from 23andme.

Variant to gene mapping
Sentinel SNPs were collected from the most recent large-scale GWAS studies. Proxies for each sentinel were queried using the proxy database, SNiPa, with the the following parameters: Genome assembly GRCh37; Variant set 1000 Genomes, Phase 3 v5; Population European; Genome annotation Ensembl 87 and r2 > 0.6. Proxy SNPs located in PIR-OCRs were then identified using bedtools intersect (bedtools v2.25.0). Analysis of contacted genes was then restricted to expressed those with a TPM > 1.

Comparison with Mouse Hypothalamic epigenetic data
We retrieved the processed data from GEO (accession GSE112125). We used liftover to convert mm9 coordinates to hg19 with the similarity cutoff -minMatch=0.1. We excluded the top 1% longest peaks for both H3K27ac ChIP-seq and ATAC-seq data. We used the R package regioneR (version regioneR 1.22.0). To perform permutation tests to determine if the accessible regions with H3K27ac+ were enriched in the dataset (10,000). We tested for overlap between the set of HN cREs and set of H3K27ac+ peaks that were significantly enriched in LepR+ neurons across conditions (FDR<0.05).

GWAS Colocalization
Summary statistics for 6 regions with overlapping associations for 3-4 input traits were imputed using FIZI (https://github.com/bogdanlab/fizi). Common variants (MAF ≥ 0.01) from the European ancestry 1000 Genomes Project v3 samples were used as a reference panel for the imputation. Default parameters were used with the exception that the minimum proportion parameter was lowered to 0.01. Standard errors and betas for the imputed SNPs were estimated using the method from https://github.com/zkutalik/ssimp_software/blob/master/extra/transform_z_to_b.R. Subsequently, HyPrColoc (https://github.com/jrs95/hyprcoloc) was used to test for colocalization across all input traits simultaneously. Separately, we tested for colocalization for each input trait genome-wide against GTEx v.7 hypothalamic eQTLs using coloc (https://github.com/chr1swallace/coloc/).

For manuscripts utilizing custom algorithms or software that are central to the research but not yet described in published literature, software must be made available to editors and reviewers. We strongly encourage code deposition in a community repository (e.g. GitHub). See the Nature Portfolio guidelines for submitting code & software for further information.

Data

Policy information about availability of data

All manuscripts must include a data availability statement. This statement should provide the following information, where applicable:

- Accession codes, unique identifiers, or web links for publicly available datasets
- A description of any restrictions on data availability
- For clinical datasets or third party data, please ensure that the statement adheres to our policy

The raw and processed ATAC-seq, Capture C, and RNA-seq data generated in this study was deposited in the gene expression omnibus (GEO) with the accession number GSE152098.

Public datasets were accessed and used in the study:
JASPAR2020: http://jaspar.genereg.net/downloads/
GTEX v7: https://gtexportal.org/home/datasets
Mouse Sorted Hypothalamic ATAC-seq and H3K27ac Chip-seq datasets: https://www.ncbi.nlm.nih.gov/geo/query/acc.cgi?acc=GSE112125
LD reference panels: https://github.com/bulik/ldsc
Molecular Signatures Database (MSigDB) v7 https://www.gsea-msigdb.org/gsea/msigdb/index.jsp
GencodeV19: https://www.gencodegenes.org/human/release_19.html
hg19/GRCh37 Genome Reference Consortium Human Reference 37 (GCA_000001405.1): http://www.ncbi.nlm.nih.gov/projects/genome/assembly/grc/

We accessed publicly available GWAS summary stats:
Age at Menarche: https://www.reprogen.org/data_download.html
Anorexia: https://www.med.unc.edu/pgc/download-results/
Bipolar Disorder: https://www.med.unc.edu/pgc/download-results/
Body Mass Index: https://portals.broadinstitute.org/collaboration/giant/index.php/GIANT_consortium_data_files
Chronotype: http://www.t2diabetesgenes.org/data/
Height: https://portals.broadinstitute.org/collaboration/giant/index.php/GIANT_consortium_data_files
Leptin Levels BMI Adjusted: https://walker05.u.hpc.mssm.edu/
Major Depressive Disorder: https://www.med.unc.edu/pgc/download-results/
Post-traumatic Stress Disorder: https://www.med.unc.edu/pgc/download-results/
Pubertal Growth: https://egg-consortium.org/
Self-reported Sleep: http://kp4cd.org/datasets/sleep
Accelerometer associated sleep traits: http://www.t2diabetesgenes.org/data/
Type II Diabetes https://cnsgenomics.com/content/data

Field-specific reporting

Please select the one below that is the best fit for your research. If you are not sure, read the appropriate sections before making your selection.

☒ Life sciences ☐ Behavioural & social sciences ☐ Ecological, evolutionary & environmental sciences

For a reference copy of the document with all sections, see nature.com/documents/nr-reporting-summary-flat.pdf

Life sciences study design

All studies must disclose on these points even when the disclosure is negative.

Sample size	An N of 4(ESCs, HPs)-6 (HNs) replicates were peformed for ATAC-seq, while RNA-seq and Promoter Capture C was performed with three replicates. These replicates originated from the same cell line (WiCell H09). Sample size either matched or exceeded the typical N for these experiments.
Data exclusions	No data was excluded from the study.
Replication	Our data was compared to publicly available datasets (GTeX) to assess its similarity with primary human tissue. We assessed reproducibility of ATAC-seq and RNA-seq data with principal component analysis. For Capture C analysis, the analysis tool (Chicago) uses the geometric mean of the read count between replicates to increase the weight of reproducible loops.
Randomization	Randomization was not a component of our experimental design. To ensure comparable results sequencing within experiments RNA-seq, ATAC-seq, Capture C respectively was performed on the same day. The majority of variation was explained by celltype mapping with the first principal component, so additional covariates were not included.
Blinding	Blinding was not performed as the data analysis pipelines required knowledge of which condition each sample belongs. Data collection was not blinded as different cells or treatments were not a component of the study, rather was stage of differentiation.

Reporting for specific materials, systems and methods

We require information from authors about some types of materials, experimental systems and methods used in many studies. Here, indicate whether each material, system or method listed is relevant to your study. If you are not sure if a list item applies to your research, read the appropriate section before selecting a response.

Materials & experimental systems

n/a	Involved in the study	
☐	☒	Antibodies
☐	☒	Eukaryotic cell lines
☒	☐	Palaeontology and archaeology
☒	☐	Animals and other organisms
☒	☐	Human research participants
☒	☐	Clinical data
☒	☐	Dual use research of concern

Methods

n/a	Involved in the study	
☒	☐	ChIP-seq
☒	☐	Flow cytometry
☒	☐	MRI-based neuroimaging

Antibodies

Antibodies used	Hoechst 33342 H3570 638181 ThermoFisher Scientific; Anti-POMC antibody ab32893 GR188614-12 Abcam; Anti-Tubulin-3 antibody 802001 B253940 BioLegend; Rabbit anti-Goat IgG, Alexa 555 A27017 TC2532792 ThermoFisher Scientific; Goat anti-Rabbit IgG, Alexa 488 A32731 SE25096 ThermoFisher Scientific
Validation	Both primary antibodies (POMC and Tubulin-3) were validated by the manufacturer

Eukaryotic cell lines

Policy information about cell lines

Cell line source(s)	WiCell Human Embryonic Stem Cell Line H9
Authentication	RNA-seq confirmed expression of stage specific marker genes during the three stages of differentiation
Mycoplasma contamination	The H9 is negative for mycoplasma. It is tested every 3 months with LookOut Mycoplasma PCR Detection Kit (MP0035-1KT)
Commonly misidentified lines (See ICLAC register)	None
